# Comparison of Three Commercially Available, AI-Driven Cephalometric Analysis Tools in Orthodontics

**DOI:** 10.3390/jcm13133733

**Published:** 2024-06-26

**Authors:** Wojciech Kazimierczak, Grzegorz Gawin, Joanna Janiszewska-Olszowska, Marta Dyszkiewicz-Konwińska, Paweł Nowicki, Natalia Kazimierczak, Zbigniew Serafin, Kaan Orhan

**Affiliations:** 1 Department of Radiology and Diagnostic Imaging, Collegium Medicum, Nicolaus Copernicus University in Torun, Jagiellońska 13-15, 85-067 Bydgoszcz, Poland; 2 Kazimierczak Private Medical Practice, Dworcowa 13/u6a, 85-009 Bydgoszcz, Poland; 3 Department of Interdisciplinary Dentistry, Pomeranian Medical University in Szczecin, 70-111 Szczecin, Poland; 4 Department of Diagnostics, Poznań University of Medical Sciences, 61-701 Poznań, Poland; 5 Department of DentoMaxillofacial Radiology, Faculty of Dentistry, Ankara University, Ankara 06500, Turkey; 6 Medical Design Application and Research Center (MEDITAM), Ankara University, Ankara 06500, Turkey; 7 Department of Oral Diagnostics, Faculty of Dentistry, Semmelweis University, 1085 Budapest, Hungary

**Keywords:** cephalometry, orthodontics, artificial intelligence, radiology

## Abstract

**Background:** Cephalometric analysis (CA) is an indispensable diagnostic tool in orthodontics for treatment planning and outcome assessment. Manual CA is time-consuming and prone to variability. **Methods:** This study aims to compare the accuracy and repeatability of CA results among three commercial AI-driven programs: CephX, WebCeph, and AudaxCeph. This study involved a retrospective analysis of lateral cephalograms from a single orthodontic center. Automated CA was performed using the AI programs, focusing on common parameters defined by Downs, Ricketts, and Steiner. Repeatability was tested through 50 randomly reanalyzed cases by each software. Statistical analyses included intraclass correlation coefficients (ICC3) for agreement and the Friedman test for concordance. **Results:** One hundred twenty-four cephalograms were analyzed. High agreement between the AI systems was noted for most parameters (ICC3 > 0.9). Notable differences were found in the measurements of angle convexity and the occlusal plane, where discrepancies suggested different methodologies among the programs. Some analyses presented high variability in the results, indicating errors. Repeatability analysis revealed perfect agreement within each program. **Conclusions:** AI-driven cephalometric analysis tools demonstrate a high potential for reliable and efficient orthodontic assessments, with substantial agreement in repeated analyses. Despite this, the observed discrepancies and high variability in part of analyses underscore the need for standardization across AI platforms and the critical evaluation of automated results by clinicians, particularly in parameters with significant treatment implications.

## 1. Introduction

Artificial intelligence (AI), a term coined in 1956 by John McCarthy, describes the ability of machines to imitate logical human behavior [1]. Recent advancements in AI technology have led to the incorporation of this technology into many fields of everyday life, including internet search engines (Google), private online assistants (Siri, Alexa), and housekeeping (iRobot). The development of AI has also made its way into the field of medicine, particularly radiology, where medical imaging in 2023 constituted approximately 85% of the FDA-approved AI programs [2]. With its significant role in imaging for treatment planning and outcome monitoring, orthodontics is one of the fields of dentistry where AI tools are being implemented most rapidly.

Since 1931, when Broadbent and Hofrath simultaneously developed a standardized method to obtain lateral cephalometric radiographs, cephalometric analysis (CA) has remained a fundamental tool used in orthodontics [3]. It allows for the precise assessment of the mandible, maxilla, and cranial base in the sagittal and vertical dimensions [4]. It involves the use of X-ray lateral cephalograms of the head and face to obtain precise linear and angular measurements between predefined landmarks. CA allows for the assessment of projected growth directions in children and adolescents, the diagnosis of malocclusion, precise treatment planning, and posttreatment evaluation. In addition to orthodontics, CA is a valuable tool in orthognathic surgery planning, ensuring precise assessment and intervention [5]. Moreover, cephalometry is used to measure changes in the pharynx and other anatomical structures in patients with obstructive sleep apnea, especially after surgical treatment [6]. Despite its high diagnostic value, CA remains a burdensome task—it is associated with the labor-intensive and time-consuming process of identifying cephalometric landmarks. Currently, the time-consuming manual measurements have been replaced with digital CA software, which facilitates quicker measurements and calculations, as well as the automatic presentation of analysis results. The digitalization of CAs has been shown to reduce the number of errors resulting from manual measurements made with a ruler and protractor [7].

The main drawbacks of manual CA are its high operator dependency and significant variability in landmark identification [8,9,10,11,12,13,14,15,16,17,18,19]. Even analyses conducted by expert orthodontists suffer from significant intrareader variability [20]. Recent advancements in artificial intelligence (AI) technology have led to the introduction of AI in many areas of dental radiology, including CA [21,22]. Compared to manual CA, automated CA is relatively stable and repeatable [23]. Several studies have reported the reliability of AI algorithms in CA, demonstrating both high diagnostic accuracy and reduced analysis time [19,24,25,26,27]. Hopefully, this approach can lead to improved workflow and productivity in dental practices.

The integration of AI in dental diagnostics has paved the way for the development of AI-based commercially available programs such as AudaxCeph (Audax, Ljubljana, Slovenia), WebCeph (Assemble Circle, Seoul, Republic of Korea), and CephX (ORCA Dental AI, Las Vegas, NV, USA). AI algorithms in CA utilize deep learning (DL) and convolutional neural networks (CNNs) to automate the identification of anatomical landmarks on radiographs. These algorithms are trained on large datasets of labeled images to learn the patterns and features associated with specific anatomical landmarks. These programs automate the identification of cephalometric points, evaluate landmarks, calculate angles and distances, and generate automated analysis reports with diagnoses. The primary advantage of such software is the ability to automatically perform CA within seconds [13]. Studies conducted to date have demonstrated a high degree of agreement between manual and AI-automated CA performed by the mentioned software [28,29,30,31]. However, to the best of our knowledge, the agreement among the results from automated CA has not been assessed.

The present study aimed to compare the agreement of the results from randomly selected, three commercially available AI tools in automated CA in one patient cohort and to assess the repeatability of the AI results. Our hypothesis is that AI-driven cephalometric analysis tools demonstrate high accuracy and interchangeability.

## 2. Materials and Methods

### 2.1. Patient Population, Sample Size Calculation

The material of this retrospective study initially consisted of 130 lateral cephalograms obtained from patients aged 12 to 20 years from the patient archives of a single, private orthodontic center. The cephalograms were selected from the initial records of new patients admitted between 2018 and 2023. After initial screening, the images were anonymized. All the cephalograms were performed on the same digital panoramic machine, Hyperion X9-Pro (MyRay, Verona, Italy). The primary indication for lateral cephalograms was orthodontic treatment planning. The selected lateral cephalograms were manually uploaded into the databases of the chosen AI programs without any image modifications (cropping, contrast adjustments, filters, etc.).

The sample size was validated according to a paper by Bonnet titled “Sample Size Requirements for Estimating Intraclass Correlations with Desired Precision” [31]. The sample size calculations were conducted using a web-based calculator (https://wnarifin.github.io/ssc/sssnsp.html, accessed on 6 May 2024). The following assumptions were made: ICC was calculated for each of the assessed parameters with a precision of 0.1, a confidence level of 90%, and a number of raters of 3.

### 2.2. Eligibility Criteria

All patients, aged 12–20 years, with lateral cephalograms acquired during the treatment planning, were consecutively enrolled in this study. Patients aged 12–20 years were selected for this study as this age range represents the common period during which orthodontic treatment is initiated and actively managed. Adolescents and young adults are the primary demographic for orthodontic interventions, making this sample representative of the population typically undergoing cephalometric analysis in clinical practice. The eligibility criteria are listed in Table 1.

### 2.3. Automatic Cephalometric Analysis

The selected lateral cephalograms were manually uploaded into the following databases of the chosen AI programs: CephX, WebCeph, and AudaxCeph. The selection of the programs included in this study was based on the commercial availability, widespread use in clinical practice, and the ability to perform fully automated cephalometric analyses. This ensures the relevance and applicability of our findings to practitioners.

The software automatically selected types of CAs and generated automatic reports. For the analysis, the common measurements from all three programs according to Downs, Ricketts, and Steiner were utilized. No manual adjustments to cephalometric landmarks were made to assess the fully automatic process of CA. The analyzed parameters are listed in Table 2.

### 2.4. Repeatability Analysis

Fifty randomly selected subjects were reuploaded as the new patients and reanalyzed by all three evaluated platforms. The intraclass correlation coefficient (ICC3) values for repeated CAs were calculated to assess the agreement among the results.

### 2.5. Statistical Analysis

The concordance of measurements of quantitative variables was assessed with ICC type 3 (according to the Shrout and Fleiss classification) [32]. The Friedman test was used to compare three or more repeated measures of quantitative variables. Paired Wilcoxon tests with Bonferroni correction served as post hoc procedures. The paired Wilcoxon test was used to compare two repeated measures of quantitative variables. The significance level for all the statistical tests was set to 0.05. All the analyses were conducted with R software, version 4.3.3.

## 3. Results

### 3.1. Population, Sample Size Calculation

After the exclusion of six cephalometric radiographs due to poor image quality (2), presence of artifacts (1), or significant double borders of the mandible (3), 124 patients (59 men, 65 women) aged 12–20 years (mean age of 14.4 years) were included in this study.

The minimum sample size calculation (*n* = 104) showed that our sample was sufficient for the validity of the results.

**Figure 1:**
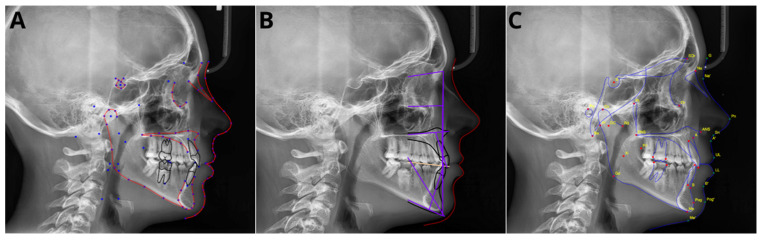
Comparison of superimposed cephalometric landmarks on sample patient: (**A**) CephX, (**B**) AudaxCeph, (**C**) WebCeph.

Figure 1 shows the cephalogram of a sample patient with superimposed cephalometric points.

### 3.2. The Results from Automated CA

The results of the analyses are presented in Table 3. Most of the analyses revealed similar mean calculation values; however, significant discrepancies were found in some of the analyzed parameters. The largest differences were demonstrated in the measurements of angular values. The greatest discrepancies were observed in the results of the angle convexity analysis, where CephX had a mean value of 176.32°, AudaxCeph had a mean value of 7.18°, and WebCeph had a mean value of 7.99°. Similar discrepancies were evident in the measurements of the occlusal plane angle, with CephX reporting a mean value of 42.8°, AudaxCeph of 6.11°, and WebCeph of 5.86°; the angle of the lower incisor (LI) to the occlusal plane—CephX 69.23°, AudaxCeph 20.31°, and WebCeph 20.62°; and the angle of the LI to the mandibular plane—CephX 87.13°, AudaxCeph 6.98°, and WebCeph 6.87°. The results of the other analyses performed showed some minor discrepancies.

Due to differences in the number of analyses performed by the AI programs, some measurements were performed only by part of the programs. A summary of the results of the analyses performed only by CephX and AudaXCeph can be found in Table 4.

### 3.3. Concordance Analysis

**Figure 2:**
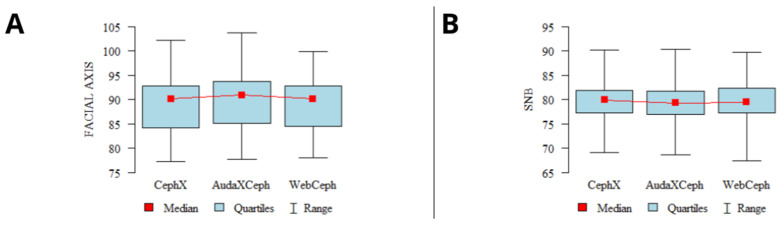
Graphs presenting sample CA parameters with excellent concordance among the 3 programs. (**A**)—facial axis; (**B**)—SNB. Mean values, 95% confidence intervals (CIs), ranges.

**Figure 3:**
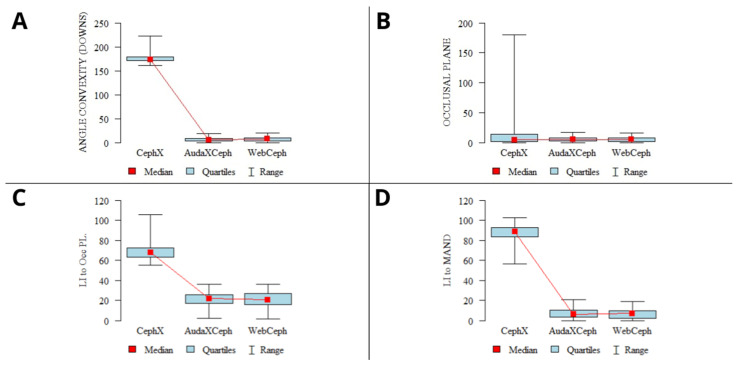
Graphs presenting sample CA parameters with poor concordance among the 3 programs. (**A**)—facial convexity angle; (**B**)—occlusal plane; (**C**)—lower incisor to occlusal plane; (**D**)—lower incisor to the mandible. Mean values, 95% confidence intervals (CIs), ranges.

The results of the concordance analysis showed good to excellent concordance of the results of the analyses for most of the parameters. However, some of the parameters showed poor and fair concordance. The detailed results of the concordance analysis of all three selected platforms are shown in Table 5. Comparisons of the results of the sample analyses showing excellent and poor concordance are shown in Figure 2 and Figure 3, respectively.

The results of the concordance analysis of the two CA platforms are shown in Table 6.

### 3.4. Repeatability Analysis

The results of the repeated analyses for 50 patients, as performed by each of the three programs, showed perfect agreement; each program returned the same results for all the repeated analyses performed.

## 4. Discussion

The present study aimed to compare the variability of the CA results of three commercially available AI-automated CA tools—CephX, WebCeph, and AudaxCeph. Our results demonstrated a high level of agreement among the AI-driven automated systems in the CA for most of the parameters evaluated. The repeatability analysis showed perfect agreement within each program, indicating that the automated systems can produce consistent results when reanalyzing the same radiographs, proving the accuracy of the algorithms. This suggests that AI-driven tools can offer a reliable alternative to traditional methods, with the added benefits of speed and consistency. Notably, significant discrepancies were observed in some angular measurements, such as angle convexity and occlusal plane angles, indicating that different methodologies were adopted by the selected platforms.

AI-driven tools offer significant advantages in CA, potentially leading to improved diagnostic accuracy, consistency in landmark identification, and a reduction in the time required for cephalometric analysis. These tools have the potential to enhance clinical decision-making, streamline workflows, and reduce the risk of human error, ultimately leading to better patient outcomes. However, the results of our study indicate significant variability in the results of several analyses among the programs. These discrepancies could be attributed to differences in algorithms, methods of evaluation, and landmark recognition capabilities across the three platforms. The highest mean differences were found in the facial angle of convexity, defined as angle convexity (CephX), angle of convexity (AudaXCeph), and N-A-Pg (WebCeph). Considering the average results of the analysis, along with their SDs, the only possibility for explaining these differences was a completely different measurement method. The inspection of the CA results of individual patients revealed that CephX indicated entirely different normal ranges than the two other programs. CephX indicated a correct value of 180 ± 5°, while the other programs indicated a value of 0 ± 5°. After eliminating CephX from this analysis, AudaXCeph and WebCeph showed very similar results, with 7.18 ± 4.53 and 7.99 ± 4.51, respectively.

The angle of convexity is usually defined as the angle formed by the intersection of two lines drawn from the most anterior point on the maxilla (Point A) and the most anterior point on the mandible (Point B) to the point on the forehead (nasion). This measurement is used to assess the relationship between the mandible and maxilla and the overall facial profile. A larger angle of convexity usually indicates a more convex facial profile, which can be associated with a protruding upper jaw or a receding lower jaw. A smaller angle indicates a straighter or more concave facial profile. The angle of convexity is a substantial parameter of CA, although its definition varies among authors. Some use the soft tissue glabellar point [33,34], the frontal point (Fr) [35], the NS point [36], or the N′ point at the depression of the nose as cranial reference points [37,38]. Godt et al. have proven that variance in the definition of landmarks used in facial convexity measurement methods can lead to significant discrepancies in the obtained angle values [39]. The differences in facial convexity automated measurement values and the normal ranges between CephX and the two other employed programs clearly show that the adapted methodology was different (Figure 3A). This example clearly shows that CephX indicated entirely different mean values and CI for angle convexity compared to AudaXCeph and WebCeph, suggesting a different measurement approach.

Similar discrepancies were found in the results of the occlusal plane calculations. These calculations were defined as the FH to the occipital plane by both CephX and AudaXCeph and as the cant of the occlusal plane by WebCeph. Again, the mean values shown by WebCeph and AudXCeph were similar, but the mean results of the CephX calculations were entirely different, yielding the results of 5.86 ± 4.05, 6.11 ± 4.01, and 42.8 ± 72.23, respectively. Notably, the results obtained by CephX showed significant variability (SD = 72.23, min = 0.13, max 179.93) compared to the significantly lower variability of the results from the rest of the analyzed platforms (Figure 3B).

The occlusal plane in the CA is an imaginary surface drawn through the incisal edges and occlusal surfaces of the teeth. It represents the mean curvature of the surface drawn rather than the actual plane. The measurement of the occlusal plane typically involves determining its angle relative to other anatomical planes or structures in the head and neck. As shown in a review by Mazurkiewicz et al., the occlusal plane might be evaluated with many different methods and devices; thus, discrepancies might be obtained [40]. The cant of the occlusal plane is defined as the vertical alignment of the teeth when there is a difference between the left and right sides. This involves either an upward or downward rotation of one side over the other in the transverse plane. The different methodologies adopted by CephX explain the discrepancies among the selected programs. However, the variability of the results from the CephX analyses (SD = 72.23, min = 0.13, max = 179.93) indicates significant discrepancies in the obtained results and raises considerable doubts about the reliability of these measurements. The results of the CephX occlusal plane measurements showed values as high as 179.93 in some patients, whereas the indicated average normal value was 9.3 ± 3.8. The same patients, assessed by AudaxCeph and WebCeph, had values of 4.68589 and 4.1, respectively. The differences shown in Figure 3B raise concerns regarding the reliability of CephX in occlusal plane measurements.

Another parameter that showed significant discrepancies was the lower incisor (LI) to the occlusal plane (Figure 3C). The mean results for CephX, AudaxCeph, and WebCeph were 69.23°, 20.31°, and 20.62°, respectively. Similarly, for the LI to the mandibular plane, the mean values were 87.13°, 6.98°, and 6.87°, respectively (Figure 3D). These significant differences in the mean values indicate that the three AI programs may have used different methods for locating the landmarks and performing the measurements. It is worth noting that these parameters are crucial in the diagnosis and treatment planning for many orthodontic cases, including those involving anterior open bite, deep overbite, and skeletal Class III malocclusion [41]. Hence, the discrepancies in these measurements among the different AI programs may lead to different diagnoses and treatment plans.

**Figure 4:**
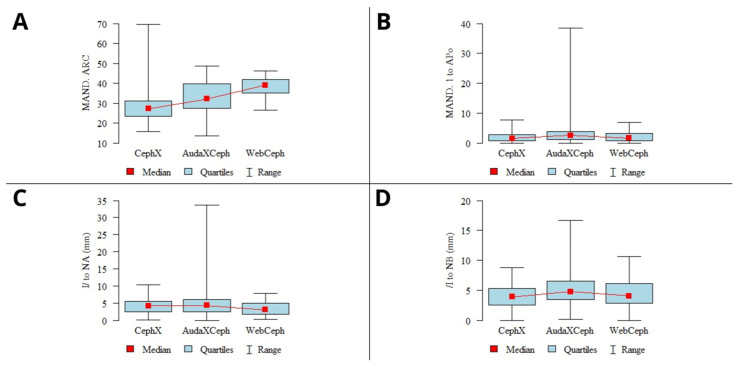
Graphs presenting sample CA parameters with poor concordance among the 3 programs. (**A**)—mandibular arc; (**B**)—MAND. 1 to APo; (**C**)—/I to NA; (**D**)—/I to NB. Mean values, 95% confidence intervals (CIs), ranges.

Despite the significant discrepancies mentioned above in the results, the vast majority of the results showed perfect agreement (ICC type 3 > 0.9). These discrepancies are in some cases related to the significantly different results of one of the three programs, likely due to different measurement methodologies. In addition to the drastically different values indicated by CephX, as discussed above, other parameters should also be mentioned. The results of the mandibular arc analysis showed variability in the mean results of all three tested platforms (Figure 4A). Moreover, the results of CephX analyses showed the highest variability, which was not comparable to that of other platforms, ranging from 15.94 to 69.51. Similarly, AudaxCeph showed high variability in MAND. 1 to APo (lower incisor—A point—pogonion) measurements, with a maximum value measured at 38.42 mm. In contrast, the maximum values indicated by CephX and WebCeph were 7.79 and 7.0, respectively (Figure 4B). The other parameters with fair agreement were the linear measurements of the distance between the lower incisor N and B or A point (/I to NA,/I to NB), with the highest variability in the AudaxCeph measurements (Figure 4C,D). However, all the indicated parameters (mandibular arc, MAND. 1 to Apo,/I to NA,/I to NB) presented 95% confidence intervals (CIs), and the standard deviations (SDs) calculated were comparable to those of other platforms, indicating likely incidental errors in landmark identification. Notably, fair concordance was found only for the distance measurements of/I to NA and/I to NB parameters, whereas the angular measurements showed perfect agreement.

To date, we have conducted two studies on CephX reliability. In the 2023 study [42], we assessed the concordance of repeated automatic CA from computed tomography (CT) images. Our results displayed good-to-excellent concordance in a large majority of measurements, except for two angular measurements: lower incisor–nasion–B-point (LI-N-B) and prosthion–nasion–A-point (Pr-N-A). The repeated analyses showed concordance results of 0 and 0.302, respectively, indicating significant discrepancies in the repeated measurements. However, it is important to note that the concordance in the repeated CA results of the current study was perfect, accounting for 1.0. These facts might demonstrate both the improvement of the automated CA module and the challenges of performing CA based on CT images. Our 2024 study [43] investigated the reliability of automated facial asymmetry assessment. Our results showed very low agreement between automatic and manual facial asymmetry measurements (ICC < 0.3). Furthermore, we found a total lack of agreement between the AI and manual asymmetry rate analyses (ICC type 3  =  0). As of the date of manuscript preparation (April 2024), CephX’s automated asymmetry assessment module is no longer available.

A recent study by Yassir et al. [44] evaluated the accuracy and reliability of WebCeph in CA. The authors reported problems with landmark identification and soft tissue delineation, and inconsistency of measurements are inherent features of the program’s automated analyses. Similar to our results, most of the discrepancies were found in the angular measurements. The 2024 study by Silva et al. [45] compared the accuracy of WebCeph and CefBot for landmark identification to that of 10 experienced readers. The authors concluded that CefBot exhibited excellent reliability and was ready for use in clinical practice, while WebCeph produced significant errors in landmark identification. A study on AudaXCeph’s tracing reliability by Ristau et al. showed that the program was not significantly different from that of experienced orthodontists [31]. However, some discrepancies in lower incisor apex measurements were found. These problems with lower incisor identification were also found in our study, yielding the high variability shown in the measurements presented in Figure 4C,D. Ribwar and Azeez compared the results of CephX and manual tracings [46]. The authors have shown that, except for several parameters, the results of the automated analysis showed high agreement with the manual method. The article concluded that CephX is adequate for clinical use.

Along with a growing body of evidence on the use of AI-automated CA in experimental studies, recent meta-analyses have been published providing a comprehensive overview of its accuracy and reliability [47,48,49,50,51,52,53]. However, most of the tested AI models were experimental and not available for common users. Furthermore, the results depend on the predefined thresholds. As expected, the accuracy sharply decreases when the threshold is lower than 2 mm [47,49,52]. A systematic review and meta-analysis by Schwendicke et al. [48] assessed the accuracy of deep learning (DL) for cephalometric landmark detection on 2D and 3D radiographs. The meta-analysis, which included 19 studies published between 2017 and 2020, revealed that DL exhibited relatively high accuracy in detecting cephalometric landmarks. However, the body of evidence suffers from a high risk of bias, highlighting the need for further studies to demonstrate the robustness and generalizability of DL for landmark detection. Rauniyar et al. [52] conducted a systematic review to determine the accuracy of identifying cephalometric landmarks using AI and compared the results with those of a manual tracing group. The review concluded that AI showed extremely positive and promising results compared to manual tracing, indicating its potential in CA. A meta-regression conducted by Serafin et al. [53] in a meta-analysis on AI-automated cephalometric landmarks indicated a significant relationship between the mean error in landmarking and the year of publication (*p*-value = 0.012). The authors concluded that the accuracy of the AI algorithms in this task has risen significantly in studies published between 2021 and 2023. These results give hope for the further development of algorithms, their refinement, and the possibility of their application in daily clinical practice.

The AI-driven CA tools used in this study are trained on large datasets of labeled images using deep learning and convolutional neural networks. These algorithms learn to identify anatomical landmarks by recognizing patterns and features from the training data. However, the specific details of these datasets and training processes are proprietary. The program’s developers refrain from providing information on this topic and treat it as a trade secret. The three AI-driven CA tools used in this study—CephX, WebCeph, and AudaxCeph—were selected based on their commercial availability, widespread use in clinical practice, and the ability to perform fully automated cephalometric analyses. These programs are among the most used AI tools in orthodontics, making this study relevant to practitioners. While other AI-driven tools exist, our access was limited to these three programs. Future studies should aim to include a broader range of AI tools to provide a more comprehensive evaluation.

A reduction in analysis time without compromising accuracy can potentially enhance productivity in orthodontic practices. Moreover, the consistency of AI-driven systems reduces the risk of human error, thus providing more standardized outcomes. However, the variations observed in certain measurements highlight the need for standardization among different AI platforms. This variation could lead to different orthodontic diagnoses and treatment plans, raising concerns about the interchangeability of these systems. It also indicates the necessity of thoroughly familiarizing oneself with the methodology of the platform used before its application. Additionally, our study demonstrated the presence of evident calculation results stemming from erroneous cephalometric landmark identification. However, practitioners must be aware of the limitations of these tools, especially regarding the discrepancies found in some angular measurements. It is imperative that practitioners critically evaluate the results from AI-driven systems and, if necessary, confirm the findings manually, especially when the measurements have significant implications for treatment decisions [54].

Despite some discrepancies, this study found high levels of agreement and repeatability in the results obtained from the AI programs for most cephalometric parameters. This suggests the potential of AI to deliver consistent and reliable analyses. The perfect repeatability of results across all evaluated programs underscores the consistency of automated analysis. AI reduces the risk of human error associated with manual CA. This standardization, coupled with high accuracy in landmark detection, can significantly improve the reliability of the assessments. In our view, our study offers valuable data that can guide the further development and refinement of AI algorithms, potentially expanding their use in clinical settings. However, this implies the need for further studies—evaluating different software with broader and more heterogeneous study groups—to provide a comprehensive evaluation of existing and future technologies. Our findings underscore the need for methodological standardization and algorithm improvement, as both factors influenced our results, indicating that current AI tools still require human supervision. Moreover, there is a vast yet insufficiently explored area of cone beam computed tomography (CBCT)-based CA. While some pilot studies have already assessed the accuracy of AI algorithms, further evaluation is still needed. Future studies should consider including a human comparison group (preferably multi-reader evaluation) to evaluate the performance of AI algorithms against experienced orthodontists. This would provide a more comprehensive understanding of the advantages and limitations of AI in cephalometry.

The findings of the present study should be interpreted considering several limitations. First, this study was retrospective in nature and relied on archived cephalograms, which might have affected the quality of some images. Moreover, there are geographic and ethnic limitations which may influence the generalizability of the results, especially in more diverse populations. Second, this study included a relatively small sample size, which may limit the generalizability of the findings. The results of the analyses were not compared to any “golden standard” based on the expert readers’ consensus; however, this was not the aim of this study. While this study demonstrates the high repeatability of AI-driven cephalometric analyses, the absence of a gold standard limits the ability to determine the most accurate tool. Future research should include manual evaluations by expert orthodontists as a benchmark to compare the AI tools, providing a comprehensive assessment of their accuracy. A further limitation of this study is the human involvement in the image selection, which implies that the process was not entirely AI-driven. Knowledge about image quality was required to exclude suboptimal radiographs, which could impact the analysis. Last, this study evaluated only three AI programs, and many other commercially available AI-driven automated CA tools were not included in the analysis.

Future studies should focus on evaluating additional AI-driven cephalometric analysis tools to provide a more comprehensive comparison. It would be beneficial to conduct studies involving larger and more diverse patient populations to enhance the generalizability of the findings. Moreover, integrating a human comparison group, including multi-reader evaluations by experienced orthodontists, could offer deeper insights into the performance of AI algorithms.

## 5. Conclusions

In conclusion, AI-driven automated CA tools can provide a quick and consistent alternative to traditional manual methods. However, significant discrepancies exist in the measurements of some parameters among different AI programs, which may potentially lead to varying diagnoses. Moreover, some parameters assessed by the selected AI platforms exhibited significant variability, indicating severe inaccuracies in landmark identification. Therefore, clinicians should be aware of these discrepancies, carefully interpreting the results of automated CA in conjunction with clinical findings and assessing the accuracy of landmark identification.

## Figures and Tables

**Table 1:** Eligibility criteria.

Criteria	Description
Inclusion Criteria	Patients aged 12–20 years
	Lateral cephalograms obtained during orthodontic treatment planning
	Good image quality without artifacts
Exclusion Criteria	Poor image quality
	Presence of artifacts or asymmetries
	Significant double borders of the mandible

**Table 2:** Categorized list of the assessed parameters as mentioned in the analyzed software.

No.	Parameter (Unified Name)	CephX	WebCeph	AudaxCeph
Downs				
1	Facial Angle	Facial Angle	FH-N-Pog	Facial Angle
2	Angle Convexity	Angle Convexity	-	N-A-Pg
3	A-B Plane	A-B Plane	NA	NPg/AB
4	Mandibular Plane	FH-GoGn	Mandibular Plane	FH/ML′
5	Y-Axis	FH-S-Gn	Y-axis	FH/Y
6	Occlusal Plane	FH-Occ Plane	Cant of the Occlusal Plane	FH/OcP
7	Upper Incisor to Lower Incisor	UI to LI	Interincisal Angle	Interincisal Angle
8	Lower Incisor to Occlusal Plane	LI to Occ Plane	Incisor Occlusal Plane Angle	−1/OcP
9	Lower Incisor to Mandibular Plane	LI to Mand	Incisor Mandibular Plane Angle	−1/ML′
10	Upper Incisor to A-Pog	UI to A-Pog	Upper Incisor to A-Pog Line	+1/APg
Ricketts				
11	Maxillary Depth	FH to N-A	NA	Maxillary Depth
12	Maxillary Height	N-PTV to A pt	NA	NA
13	SN to Palatal Plane	SN TO PALATAL PLANE	NA	NA
14	Facial Depth	FH to N-Pog	Facial Depth	Facial Depth
15	Facial Axis	Na-Ba to PTV-Gn pt	Facial Axis	Facial Axis
16	Facial Taper	Na-Gn-Go	Facial Taper	Facial Taper
17	Mandibular Plane (Ricketts)	FH-GoGn	Mandibular Plane Angle (Ricketts)	FMA
18	Corpus Length	Xi to Pm	NA	Corpus Axis
19	Mandibular Arc	DC-Xi to Xi-Pm	Mandibular Arc	Mandibular Arc
20	Point A Convexity	A to N-Pog	Convexity of Point A	Convexity
21	Lower Facial Height	ANS-Xi-Pog	Denture Height (Lower Facial Height)	Lower Facial Height
23	Maxillary Incisor to A-Po	MAX.1 to APo	NA	NA
24	Upper Molar to PTV	MAX.6 to PTV	Upper Molar to PtV	NA
25	Mandibular Incisor to A-Po	MAND. 1 to APo	L1 to A-Pog (mm)	Lower 1 to APg (mm)
26	Hinge Axis Angle	DC-Go-LI	NA	NA
27	Maxillary Incisor to Mandibular Incisor	MAX.1 to MAND.1	Intercisal Angle	Interincisal Angle
28	Overjet	Overjet	NA	NA
29	Overbite	Overbite	NA	NA
31	Upper Lip to E-Line	Upper Lip to E-Line	NA	NA
32	Lower Lip to E-Line	Lower Lip to E-Line	Lower Lip to E-Plane	Li/E-Line
Steiner				
33	SNA	SNA	SNA	Angle SNA
34	SNB	SNB	SNB	Angle SNB
35	ANB	ANB	ANB	ANB
36	Maxillary Incisor to NA (deg)	I/to NA	U1 to NA (deg)	+1/NA
37	Maxillary Incisor to NA (mm)	I/to NA	U1 to NA (mm)	+1i/NA
38	Mandibular Incisor to NB (deg)	/I to NB	L1 to NB (deg)	−1/NB
39	Mandibular Incisor to NB (mm)	/I to NB	L1 to NB (mm)	−1i/NB
40	Interincisal Angle	Interincisal Angle	Interincisal Angle	Interincisal Angle
41	Occlusal Plane to SN	Occ to SN	Occlusal Plane to SN Angle	SN/OcP
42	Mandibular Plane to SN	GOGN-SN	Mandibular Plane Angle (Go-Gn to SN)	SN/GoGn
43	Pogonion to NB	Pog to NB	NA	Pg/NB

NA—not assessed.

**Table 3:** Summary of the mean results of analyses performed by all three selected CA platforms.

Parameter	Software	Mean	SD	Median	Min	Max	Q1	Q3	*p*
FACIAL ANGLE	CephX-A	89.54	3.64	89.09	82.68	101.20	87.08	91.62	*p* = 0.001 * B.A > C
	AudaXCeph-B	89.46	3.46	89.26	81.30	103.31	87.55	91.05	
	WebCeph-C	88.77	3.43	89.03	81.78	99.89	86.49	90.56	
ANGLE CONVEXITY (DOWNS)	CephX-A	176.32	10.41	174.00	161.78	222.66	171.13	179.30	*p* < 0.001 * A > C > B
	AudaXCeph-B	7.18	4.53	6.28	0.66	19.68	3.77	9.69	
	WebCeph-C	7.99	4.51	7.88	0.50	20.43	4.10	10.96	
MAND. PLANE (DOWNS)	CephX-A	24.90	5.61	23.23	13.63	37.34	21.21	29.57	*p* < 0.001 * A > B.C
	AudaXCeph-B	22.49	5.79	22.01	12.12	34.32	17.30	27.68	
	WebCeph-C	22.05	6.31	21.01	10.55	33.25	17.13	27.44	
Y-AXIS	CephX	58.13	4.00	58.18	49.52	65.30	56.03	61.06	*p* = 0.064
	AudaXCeph	57.74	3.90	57.64	48.28	66.25	55.41	60.21	
	WebCeph	58.02	3.93	58.20	48.75	65.03	55.67	61.61	
OCCLUSAL PLANE	CephX-A	42.80	72.23	5.53	0.13	179.93	1.65	13.98	*p* = 0.01 * A > C
	AudaXCeph-B	6.11	4.01	5.38	0.32	17.61	3.21	8.38	
	WebCeph-C	5.86	4.05	5.27	0.09	16.65	2.50	8.48	
UI to LI	CephX-A	130.06	11.10	129.31	103.04	167.22	123.03	135.18	*p* = 0.029 * B > A
	AudaXCeph-B	131.39	11.25	130.48	106.21	162.23	123.94	138.08	
	WebCeph-C	130.29	10.96	129.28	105.95	160.59	123.05	137.04	
LI to Occ PL.	CephX-A	69.23	8.33	67.93	55.17	105.49	63.26	72.47	*p* < 0.001 * A > B.C
	AudaXCeph-B	20.31	7.30	21.70	2.32	36.02	17.22	25.68	
	WebCeph-C	20.62	7.52	20.76	1.88	36.08	16.00	27.19	
LI to MAND	CephX-A	87.13	7.93	88.60	56.63	102.33	83.69	92.10	*p* < 0.001 * A > C.B
	AudaXCeph-B	6.98	4.72	6.35	0.09	21.00	3.53	9.73	
	WebCeph-C	6.87	4.48	7.39	0.13	18.85	2.79	9.56	
UI to A-Pog	CephX-A	6.19	2.97	6.17	0.31	14.71	3.88	8.38	*p* < 0.001 * A > B > C
	AudaXCeph-B	5.94	2.83	5.73	0.03	12.21	3.99	8.48	
	WebCeph-C	5.48	2.74	5.46	0.82	13.89	3.66	7.58	
FACIAL DEPTH	CephX-A	89.55	3.42	89.42	82.68	98.53	87.10	91.91	*p* = 0.003 * A.B > C
	AudaXCeph-B	89.35	3.07	89.30	81.30	97.54	87.45	91.15	
	WebCeph-C	88.76	3.15	89.06	81.78	97.53	86.50	90.60	
FACIAL AXIS	CephX-A	89.25	5.39	90.29	77.27	102.22	84.19	92.59	*p* < 0.001 * B > A.C
	AudaXCeph-B	90.14	5.44	90.92	77.84	103.75	85.16	93.68	
	WebCeph-C	89.17	5.27	90.18	78.05	99.90	84.45	92.83	
FACIAL TAPER	CephX-A	66.91	4.74	67.08	55.14	74.20	64.08	70.52	*p* < 0.001 * C > A.B
	AudaXCeph-B	66.90	5.06	66.79	53.22	75.07	64.10	70.57	
	WebCeph-C	72.23	6.61	72.41	53.43	84.50	69.38	76.56	
MAND. PLANE (RICKETTS)	CephX-A	24.54	5.65	22.61	13.63	37.34	20.91	29.02	*p* < 0.001 * B > A > C
	AudaXCeph-B	23.75	5.74	22.95	13.50	35.81	18.75	28.96	
	WebCeph-C	21.89	6.23	20.76	10.55	33.25	16.70	27.44	
MAND. ARC	CephX-A	28.05	7.82	28.35	15.94	69.51	23.85	31.75	*p* < 0.001 * C > B > A
	AudaXCeph-B	33.06	8.45	32.38	13.76	48.67	27.53	39.69	
	WebCeph-C	37.76	5.21	39.13	26.51	46.27	35.12	41.85	
A pt. CONVEXITY	CephX-A	3.46	2.14	3.34	0.17	9.28	2.26	4.42	*p* = 0.022 * B > A
	AudaXCeph-B	3.68	4.22	2.85	0.27	29.82	1.78	4.27	
	WebCeph-C	3.60	1.96	3.66	0.24	8.55	2.18	4.86	
LOW.FACE.HEIGHT	CephX-A	41.15	5.26	40.20	29.38	51.25	37.05	45.31	*p* < 0.001 * C > B > A
	AudaXCeph-B	43.56	6.67	42.71	32.35	56.68	37.71	48.96	
	WebCeph-C	44.23	6.08	43.14	31.43	55.66	39.17	49.21	
MAND.1 to APo	CephX-A	2.13	1.79	1.48	0.11	7.79	0.80	3.04	*p* < 0.001 * B > C.A
	AudaXCeph-B	3.31	5.26	2.64	0.04	38.42	1.19	3.96	
	WebCeph-C	2.11	1.73	1.73	0.07	7.00	0.74	3.26	
MAX.1 to MAND.1	CephX-A	130.06	11.10	129.31	103.04	167.22	123.03	135.18	*p* = 0.029 * B > A
	AudaXCeph-B	131.39	11.25	130.48	106.21	162.23	123.94	138.08	
	WebCeph-C	130.29	10.96	129.28	105.95	160.59	123.05	137.04	
LOWER LIP to E-LINE	CephX	2.60	2.11	1.90	0.02	9.78	1.04	4.12	*p* = 0.981
	AudaXCeph	2.90	2.17	2.58	0.15	8.67	0.91	4.28	
	WebCeph	2.67	2.01	2.50	0.03	6.98	1.08	4.13	
SNA	CephX-A	82.89	3.49	83.21	74.48	89.74	81.06	85.23	*p* = 0.039 * A > B
	AudaXCeph-B	82.38	3.67	82.71	73.90	89.71	79.73	84.83	
	WebCeph-C	82.70	3.50	83.10	73.84	89.48	80.33	85.22	
SNB	CephX-A	79.59	4.29	80.00	69.16	90.13	77.36	81.75	*p* = 0.035 * A > C.B
	AudaXCeph-B	79.32	4.36	79.37	68.70	90.36	76.98	81.79	
	WebCeph-C	79.09	4.19	79.48	67.43	89.65	77.34	82.40	
ANB	CephX-A	4.08	2.25	4.06	0.28	9.97	2.38	5.56	*p* = 0.008 * C > B
	AudaXCeph-B	3.85	2.18	3.83	0.31	10.08	2.25	5.35	
	WebCeph-C	4.35	2.19	4.56	0.03	8.80	2.73	5.79	
I/to NA (deg)	CephX-A	23.32	8.38	22.00	5.76	44.38	18.71	28.05	*p* < 0.001 * A.C > B
	AudaXCeph-B	20.83	8.80	20.51	1.19	41.24	16.04	25.43	
	WebCeph-C	21.99	8.45	21.23	0.55	39.50	17.92	27.02	
I/to NA (mm)	CephX-A	4.05	2.34	4.06	0.13	8.90	2.46	5.56	*p* = 0.011 * A.B > C
	AudaXCeph-B	4.64	4.78	3.59	0.06	33.65	2.41	5.94	
	WebCeph-C	3.48	2.15	3.09	0.34	7.82	1.85	4.98	
/I to NB (deg)	CephX	22.85	6.46	24.65	2.10	31.14	19.14	27.23	*p* = 0.118
	AudaXCeph	24.03	6.88	25.60	6.51	34.92	19.68	29.18	
	WebCeph	23.58	6.88	24.68	4.21	35.43	19.45	28.45	
/I to NB (mm)	CephX-A	3.89	2.17	3.85	0.05	8.76	2.48	5.18	*p* < 0.001 * B > C > A
	AudaXCeph-B	4.98	2.88	4.62	0.19	16.66	3.18	6.44	
	WebCeph-C	4.45	2.33	4.07	0.03	10.69	2.86	6.10	
I/to/I	CephX-A	130.06	11.10	129.31	103.04	167.22	123.03	135.18	*p* = 0.029 * B > A
	AudaXCeph-B	131.39	11.25	130.48	106.21	162.23	123.94	138.08	
	WebCeph-C	130.29	10.96	129.28	105.95	160.59	123.05	137.04	
Occ to SN	CephX-A	12.59	5.10	11.66	3.71	27.11	8.96	15.08	*p* < 0.001 * B > C > A
	AudaXCeph-B	14.79	4.87	14.31	5.49	24.36	11.23	17.98	
	WebCeph-C	14.04	4.68	13.40	2.42	23.03	10.61	17.53	
GOGN-SN	CephX-A	36.22	6.35	35.59	25.68	47.29	30.94	41.98	*p* < 0.001 * A > B > C
	AudaXCeph-B	30.40	6.91	30.40	18.48	41.91	24.65	36.83	
	WebCeph-C	29.33	6.85	28.25	16.41	42.20	23.76	35.33	

*p*—Friedman test + post hoc analysis (paired Wilcoxon tests with Bonferroni correction). * statistically significant (*p* < 0.05).

**Table 4:** Summary of the mean results of analyses performed by two of the selected platforms (CephX and AudaXCepx).

Parameter	Measurement	Mean	SD	Median	Min	Max	Q1	Q3	*p*
A-B PLANE	CephX	6.84	3.43	7.1	0.14	15.35	4.23	9.68	*p* < 0.001 *
	AudaXCeph	9.79	5.41	9.43	0.95	23.64	5.8	14.06	
MAX DEPTH	CephX	91.89	2.97	91.47	86.54	99.13	89.98	94.02	*p* = 0.004 *
	AudaXCeph	91.31	2.99	91.41	83.84	97.46	89.52	93.06	
CORPUS LENGTH	CephX	73.77	6.5	73.43	39.67	88.53	70.22	78.12	*p* < 0.001 *
	AudaXCeph	72.14	37.69	67.88	59.11	391.47	64.36	70.01	
POG to NB	CephX	1.96	1.59	1.74	0	6.69	0.62	3.01	*p* < 0.001 *
	AudaXCeph	2.46	1.66	2.21	0.18	6.24	1.24	3.47	

*p*—Friedman test + post hoc analysis (paired Wilcoxon tests with Bonferroni correction). * statistically significant (*p* < 0.05).

**Table 5:** Results of the concordance analysis of all three CA platforms.

Parameter	ICC	95% CI		Agreement (Cicchetti)	Agreement (Koo and Li)
FACIAL ANGLE	0.910	0.864	0.943	Excellent	Excellent
ANGLE CONVEXITY (DOWNS)	0.075	−0.073	0.254	Poor	Poor
MAND. PLANE (DOWNS)	0.903	0.854	0.939	Excellent	Excellent
Y AXIS	0.927	0.889	0.954	Excellent	Excellent
OCCLUSAL PLANE	0.000	−0.137	0.172	Poor	Poor
UI to LI	0.942	0.913	0.963	Excellent	Excellent
LI to Occ PL.	0.000	−0.137	0.172	Poor	Poor
LI to MAND	0.388	0.222	0.553	Poor	Poor
UI to A-Pog	0.922	0.881	0.951	Excellent	Excellent
FACIAL DEPTH	0.898	0.845	0.937	Excellent	Good
FACIAL AXIS	0.975	0.960	0.984	Excellent	Excellent
FACIAL TAPER	0.789	0.692	0.865	Excellent	Good
MAND. PLANE (RICKETTS)	0.901	0.849	0.938	Excellent	Excellent
MAND. ARC	0.510	0.348	0.659	Fair	Fair
A pt. CONVEXITY	0.532	0.373	0.676	Fair	Fair
LOW.FACE.HEIGHT	0.947	0.918	0.967	Excellent	Excellent
MAND. 1 to APo	0.416	0.246	0.582	Fair	Poor
MAX.1 to MAND.1	0.942	0.913	0.963	Excellent	Excellent
LOWER LIP to E-LINE	0.799	0.705	0.871	Excellent	Good
SNA	0.897	0.843	0.935	Excellent	Good
SNB	0.968	0.950	0.981	Excellent	Excellent
ANB	0.908	0.860	0.943	Excellent	Excellent
I/to NA (deg)	0.918	0.873	0.949	Excellent	Excellent
I/to NA (mm)	0.494	0.331	0.646	Fair	Poor
/I to NB (deg)	0.938	0.903	0.961	Excellent	Excellent
/I to NB (mm)	0.745	0.633	0.834	Good	Fair
I/to/I	0.942	0.913	0.963	Excellent	Excellent
Occ to SN	0.903	0.853	0.940	Excellent	Excellent
GOGN-SN	0.931	0.893	0.957	Excellent	Excellent

ICC—interclass correlation coefficient, CI—confidence interval.

**Table 6:** Results of the concordance analysis of two of the selected platforms (CephX and AudaxCeph).

Parameter	ICC	95% CI		Agreement (Cicchetti)	Agreement (Koo and Li)
A-B PLANE	0.798	0.698	0.868	Excellent	Good
MAX DEPTH	0.844	0.764	0.899	Excellent	Good
CORPUS LENGTH	0.123	−0.106	0.339	Poor	Poor
POG to NB	0.945	0.914	0.965	Excellent	Excellent

ICC—interclass correlation coefficient, CI—confidence interval.

## Data Availability

The data are available on request.
